# Differences in cortical response to acupressure and electroacupuncture stimuli

**DOI:** 10.1186/1471-2202-12-73

**Published:** 2011-07-27

**Authors:** Thomas Witzel, Vitaly Napadow, Norman W Kettner, Mark G Vangel, Matti S Hämäläinen, Rupali P Dhond

**Affiliations:** 1 Harvard Medical School, Martinos Center for Biomedical Imaging, Charlestown, MA 02129 USA; 2 Logan College of Chiropractic, Department of Radiology, Chesterfield, MO 63017 USA

## Abstract

**Background:**

FMRI studies focus on sub-cortical effects of acupuncture stimuli. The purpose of this study was to assess changes in primary somatosensory (S1) activity over the course of different types of acupuncture stimulation. We used whole head magnetoencephalography (MEG) to map S1 brain response during 15 minutes of electroacupuncture (EA) and acupressure (AP). We further assessed how brain response changed during the course of stimulation.

**Results:**

Evoked brain response to EA differed from AP in its temporal dynamics by showing clear contralateral M20/M30 peaks while the latter demonstrated temporal dispersion. Both EA and AP demonstrated significantly decreased response amplitudes following five minutes of stimulation. However, the latency of these decreases were earlier in EA (~30 ms post-stimulus) than AP (> 100 ms). Time-frequency responses demonstrated early onset, event related synchronization (ERS), within the gamma band at ~70-130 ms and the theta band at ~50-200 ms post-stimulus. A prolonged event related desynchronization (ERD) of alpha and beta power occurred at ~100-300 ms post-stimulus. There was decreased beta ERD at ~100-300 ms over the course of EA, but not AP.

**Conclusion:**

Both EA and AP demonstrated conditioning of SI response. In conjunction with their subcortical effects on endogenous pain regulation, these therapies show potential for affecting S1 processing and possibly altering maladaptive neuroplasticity. Thus, further investigation in neuropathic populations is needed.

## Background

Acupuncture is a component of Traditional Chinese Medicine (TCM) most often characterized by stimulation of specific body sites with sharp 'insertive' needles. However, blunt 'non-insertive' needles (Teishein needles) may also be used to apply rapid percussive pressure, specifically tapping, without penetrating the skin, akin to 'acupressure'. Modern insertive acupuncture frequently involves electrical stimulation applied directly to inserted needles (electroacupuncture). In a typical clinical acupuncture session, a patient may be stimulated manually or electrically at one or more body sites for > 10 minutes. During this time the patient is often left alone to 'relax' without additional sensory stimuli and there is no requirement for focused attention. Recently, acupuncture has been gaining popularity in the West as a complementary therapy and much research is aimed at elucidating its neural correlates.

Currently, fMRI data show that insertive acupuncture activates subcortical brain areas which are implicated in endogenous anti-nociception [for review 1]. Numerous animal studies show that acupuncture analgesia is mediated by opiodergic and/or monoaminergic neurotransmission involving the brainstem, thalamus and hypothalamic-pituitary-adrenal axis [2-7]. This has also been evidenced in humans using PET [8]. In the case of painful needling, afferent spinal gating and diffuse noxious inhibitory control (DNIC) may provide short-term analgesia [9].

*However, these are not the only ways acupuncture may exert effects on the body*. Interestingly, even though fMRI investigations have found greater subcortical responses for insertive acupuncture vs. non-insertive acupressure-like tapping they have shown stronger S1 response to the latter [10-13]. Recent neuroimaging data also suggest that the regular afferent stimulation provided by acupuncture may affect neuroplasticity in S1 cortex [14,15]. Thus, further investigation of the cortical signatures for electroacupuncture (EA) as well as acupressure stimulation would be useful. Since S1 neural activity occurs on a millisecond timescale, magnetoencephalography (MEG) may be used to non-invasively study these cortical responses.

In the present study, we used MEG to spatiotemporally map somatosensory cortical response to different types of acupuncture, electroacupuncture (EA) and acupressure (AP). Forearm acupoints were chosen based on ease of access and because MEG is biased towards superficial brain activity [16] making them easier to localize than leg acupoints. *Importantly, we sought to mimic clinical acupuncture intervention procedures as much as possible*. To do this we *intentionally *lacked control for attention and did *not *utilize concurrent sensory (i.e. non-EA or non-AP) stimuli. Furthermore, both EA and AP were given with clinically relevant parameters, i.e. 2 Hz stimulus rate and > 10 min. duration. Finally, since acupoint specificity remains debatable [17] and may be exemplified predominantly as differences in somatotopic localization, we tested EA and AP at the same forearm acupoints.

## Results

In the present study 16 healthy, right handed, subjects were given EA and non-invasive AP each at a low frequency (2 Hz) for 15 minutes while MEG was recorded.

### SI Sources and Evoked Timecourse Modulation

**Figure 1:**
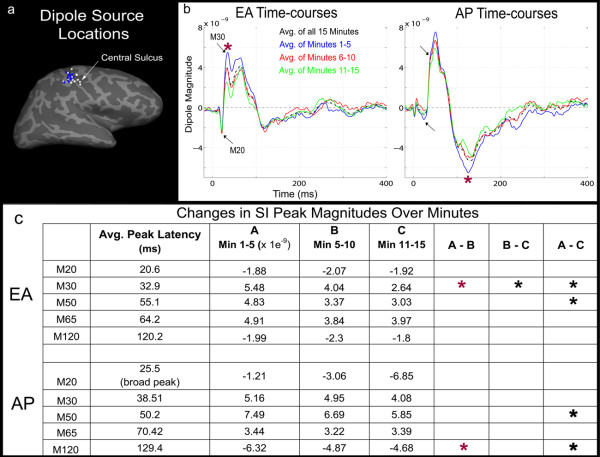
**Evoked S1 responses to Electroacupuncture (EA) and Acupressure (AP)**: **(a) **For all subjects, the primary sources localized to the contralateral SI for EA (white dots) and AP (blue dots). Sources are shown as closest points on the cortex reconstructed from subjects' individual MRIs and morphed to an average inflated brain surface (sulci/gyri are dark/light gray respectively). **(b) **Average time-courses for all trials of EA and AP (black, dashed lines) demonstrated analogous M20, M30, M50/M60 and M120 (those of AP being slightly delayed). Five minute sub-averages (blue, red and green lines) show attenuation of peak-to-peak amplitude over the course of the run. **(c) **Peaks demonstrating significant decreases (p < 0.01) following 5 minutes of stimulation are marked by a red "*" For EA this was the M30 peaks and for AP the 130 ms peak. Significant differences occurring after > 10 minutes stimulation were seen for the M30 and M50 with EA and with the M50 and M120 with AP.

For both EA and AP, the primary sources of MEG activity in each subject, localized to the contralateral primary somatosensory (SI) cortex, roughly area 3b (Figure 1a). EA/AP sources neighbored one another with AP sources mapping slightly dorsal to EA in most subjects. The average response for 15 minutes of EA stimulation (Figure 1b; black time-courses) first peaked at 20.6 ms post-stimulus and then at 32.9 ms (black arrows). The spatiotemporal characteristics of these responses were similar to the M20 and M30 components evoked by median nerve stimulation [18,19]. For AP, initial peaks 25.5 ms and 38.5 ms shown (black arrows) were similar in orientation but delayed compared to EA. Both conditions also demonstrated peaks at ~50 ms and ~65 ms, with those for AP again slightly delayed. Wide, long-latency, peaks occurred for both stimuli; at 120.2 ms for EA and 129.4 ms for AP. To determine if/how evoked SI responses were modulated during both 15 minute runs of EA and AP stimulation, individual data were divided into three, 5 minute sub-averages (Figure 1b; blue, red and green time-courses). These sub-averages showed attenuation of peak-to-peak amplitude for progressively later trials. Significant decreases in peak amplitude (Figure 1c) following 5 minutes of stimulation (red (*) paired t-tests, df(15), p < 0.01) were found for EA at 30 and 50 ms and for AP at 50 and 130 ms.

### Oscillatory Responses during EA and AP Stimulation

**Figure 2:**
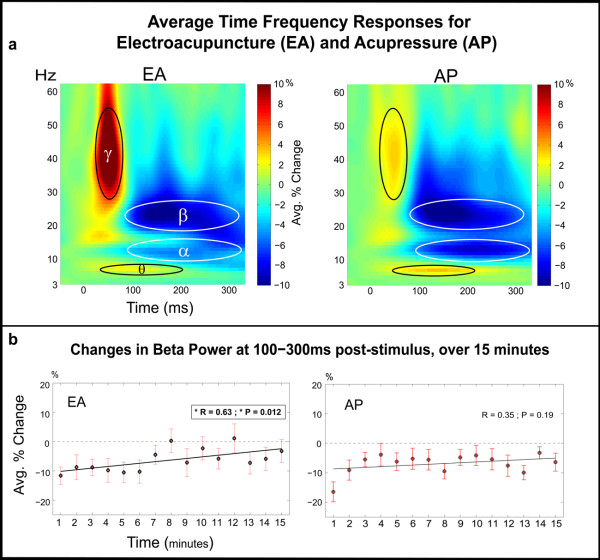
**S1 Oscillatory Activity: (a) **Post-stimulus time-frequency representations showed an early ~20-70 ms low-gamma (**γ**) ERS. This was followed by a prolonged ERD in alpha (**α**) and beta (**β**) power at ~100-300 ms post-stimulus. There was a simultaneous theta (**θ**) ERS at ~50-200 ms. **(b) **Beta ERD at 100-300 ms was calculated for each minute of stimulation and demonstrated a trend for decrease over the course of EA stimulation. Similar calculations were made for other frequency bands but did not show significance at the same p < 0.01 level.

Time frequency response (TFR) analysis demonstrated early and late modulation of oscillatory activity in S1 (Figure 2a ). Early response included low gamma (~30-50 Hz, γ) ERS from ~20-70 ms post-stimulus and theta (~6-8 Hz, θ) ERS at ~50-200 ms. This was followed by a prolonged decrease in mu frequency power, centered at alpha (~8-13 Hz, α) and beta (~15-30 Hz, β) at ~100-300 ms post-stimulus. No significant differences were found between EA and AP in any of these ranges (paired t-test, p < .01).

To determine how/if oscillatory response changed over time, EA and AP data were segmented into fifteen separate averages, each 1 minute in length. The relative power between 100-300 ms post-stimulus for each minute and frequency band was assessed. Interestingly, over the course of EA the magnitude of beta ERD appeared to decrease (Figure 2b ). A regression and goodness of fit test was performed, demonstrating a linear trend for EA (R = 0.62 and p = 0.01). However for AP, no significant linear trend was found (R = 0.35 and p = 0.19). These same calculations were performed for the alpha, theta and gamma band (results available upon request) but no trends at the same significance level were seen. However, when comparing only the first and last minute, there appears to be some decrease in beta ERD for AP also (paired t-test df(15), p < 0.03). This reduction appears qualitatively as rapid reduction in induced response during the first 3-4 minutes of stimulation with no significant trend during later minutes. For EA, this same comparison was just under significance (paired t-test df(15), p < 0.07).

## Discussion

In the present experiment we sought to spatiotemporally map somatosensory response to different types of acupuncture, EA and AP.

### Basic Response to EA and AP

EA and AP responses most consistently mapped to contralateral SI (Figure 1a ). However, AP produced a smaller/broader M20 and slightly delayed M30 like responses (Figure 1b ). Thus, with EA, underlying skin receptors/afferents were recruited simultaneously, while gradual skin indentation during AP likely produced temporal dispersion of afferent sensory signals. EEG studies have found that early SEPs (< 40 ms) [20] and SEFs [21] to mechanical stimulation are often delayed and less pronounced than those from electrical stimulation. Furthermore, AP evoked stronger brain response than EA, particularly at long latencies (> 80 ms). This may be explained by differences in the number and/or type of somatosensory fibers recruited. Although, both stimuli were carried at least in part by fast Aβ sensory fibers, it is possible that the relatively larger surface area of the blunt acupressure needle tip activated *more *superficial sensory fibers than did EA. Differences in the magnitude of brain response to acupuncture vs. non-invasive control stimulation have also been noted in fMRI studies [10,13].

Time frequency response for EA (and AP) demonstrated early onset ERS at ~20-70 ms in the gamma range (Figure 2a ). Previous work utilizing visual tasks suggests that early gamma activity may reflect "stimulus selection" or local "binding" of attributes related to initial stimulus perception [22]. Similarly, the early latency of gamma activity in this study, suggests it may support stimulus selection in SI cortex. Theta ERD which began early (~50 ms) but continued into longer latencies (~200 ms) may support general aspects of stimulus prediction [23]. Prolonged mu ERD ~100-300 ms, as in other tactile MEG studies, is consistent with the presence of afferent somatosensory input [24,25]. To understand if/how brain activity is modulated during the course of acupuncture and acupressure we assessed changes in the EA and AP response over the course of 15 minutes.

### Changes SI activity during EA and AP

Cortical activity is thought to be in a constant state of use dependant fluctuation which may exist with or without higher order processes such as attention [26]. In our experiment, brain activity to EA and AP changed over many minutes, indicating habituation or conditioning of response.. Although, the first cortical response (M20) maintained a consistent amplitude, evoked responses at > 30 ms post-stimulus were significantly attenuated after 5 minutes of EA (Figure 1b). This was accompanied by a gradual decrease in beta ERD at ~100-300 ms post-stimulus (Figure 2b) but not in the theta, alpha or gamma frequency bands (analysis available on request). Suggesting that in the domain of sensory conditioning, local beta band activity may be most important.

Furthermore, the earliest S1 evoked response (M20) represents bottom-up signals propagating from layer 4 (the primary site of thalamic afferent input) to layers 2/3 in cortex [27-30]. The post-stimulus latency of repetition induced decreases (i.e. occurring *after *20 ms) suggests they may be linked to top-down cortical mechanisms including decreased attention [18,31]. *It should be noted that, in the present experiment we aimed to mimic a clinical acupuncture setting by maintaining continuous (≥ 10 min.) acupoint stimulation and not actively engaging subjects in a concurrent distractor task*. Similarly, mu ERD is also attenuated when subjects are less attentive to stimulus events [32-34]. Here, repetition induced decreases similar to those seen in somatosensory attentional paradigms likely reflects stimulus recognition and possibly, modification of somatosensory memories.

Recent data suggest acupuncture stimulation may induce beneficial cortical plasticity [14,35] in neuropathic states. For example, following five-weeks of acupuncture treatment on the affected arm, carpal tunnel syndrome patients demonstrated clinical improvement (decreased parasthesias) and less S1 fMRI hyperactivation compared with pretreatment baseline [15]. Specifically it is hypothesized that the regular afferent input provided by acupuncture acts as a conditioning stimulus to counteract local symptoms of spontaneous activity occurring with parasthesias. It is well known that peripheral nerve lesions induce cortical reorganization [36-38] which may correlate with symptoms of hyperalgesia and pain [39]. However, such maladaptive plasticity may be reduced (or prevented) with therapy involving sensorimotor and/or visual feedback [40,41]. However, even in the absence of injury, somatosensory conditioning in the form of electrotherapy may help counteract normal age related sensorimotor decline [42].

In the electrophysiological domain, somatosensory conditioning may be marked not only by decreased ERF response but by changes in S1 beta activity over time. However, this is highly speculative and data from clinical populations is required. Although exposure related changes are also seen for AP the timing of evoked responses and the lack of change in the spectral domain suggests that a more regulated and sharper stimulus (EA) compared to a temporally diffuse mechanical stimulus (AP). Thus, although both may prove useful somatosensory conditioning stimuli, it is possible that EA may prove to be a better conditioning stimulus for neuropathic conditions where maladaptive central plasticity may be maintained by diffuse unregulated (spontaneous) afference - i.e. paresthesias in CTS. However, this is again speculative and requires testing in clinical populations.

## Conclusion

We used MEG to map somatosensory brain response during 15 minutes of electroacupuncture and acupressure. ERF's to EA and AP most consistently localized to contralateral SI. However, AP differed in its temporal dynamics showing delayed response peaks, consistent with mechanical stimulation. Both EA and AP demonstrated significantly decreased response amplitudes, following five minutes of stimulation. However, the latency of these decreases occurred much earlier in EA (~30 ms post-stimulus) than AP (> 100 ms). Time-frequency responses demonstrated early onset, event related synchronization (ERS), within the gamma band at ~70-130 ms and the theta band at ~50-200 ms post-stimulus. A prolonged event related desynchronization (ERD) of alpha and beta power occurred at ~100-300 ms post-stimulus. There was decreased beta ERD at ~100-300 ms over the course of EA, but not with AP. The precise timing provided by EA stimulation supports its role as a conditioning stimulus which may be used to affect maladaptive neuroplasticity.

## Methods

### Subjects and Experimental Paradigm

Imaging data were collected from 16 healthy subjects between 20-44 years of age. This study was in compliance with the Helsinki Declaration. All subjects gave written consent and were screened to assure their safety and compatibility for MEG and MRI recordings. During the MEG experiment subjects were seated with their head in the dewar and instructed to fixate on a centrally presented "+" sign. Both EA and AP consisted of 15 minutes continuous low-frequency (2 Hz) stimulation given on the left medial forearm at acupoint PC-6 (pericardium-6, neiguan). Forearm acupoints were used primarily based on ease of access and because MEG is biased towards superficial brain activity. Thus, SI responses for points on the forearm are more accurately mapped with MEG than those of the leg which are located medially in the brain [16]. All acupuncture was performed by the same licensed acupuncturist and the order of EA and AP runs was randomized across subjects. During each 10 minute rest run there was no acupuncture intervention and subjects were required to sit quietly. Rest runs were used to reduce residual sensations between acupuncture runs. Subjects wore earplugs throughout the experiment to attenuate any sounds heard from outside the MEG room or from stimulation equipment.

### Acupuncture Procedures

Participants were told they would receive "two different types" of acupuncture and were prevented from viewing insertion and stimulation procedures through the use of an opaque screen. During the experiment subjects wore a plastic brace on their forearm to prevent fist clenching and excessive hand movement. A rectangular opening over the ventral forearm provided access to acupoints. The EA run involved needle insertion and manipulation, to elicit *deqi^1 ^*sensation, after which electrical current was delivered. Current amplitude was set to the level at which subjects indicated feeling a "strong but not painful" sensation. Current was delivered as a monophasic rectangular, constant-current pulse (pulse width: 0.2 ms at 2 Hz) using a GRASS stimulator (S88 Dual Output Square Pulse Stimulator, Grass Telefactor, West Warwick, RI). To most closely match active stimulation in the EA run, the AP procedure involved AP *insertion *followed by *stimulation*. During AP insertion, subjects were palpated near the acupoint to mimic acupoint localization (as with EA). Then needle insertion and manipulation was simulated with a previously validated technique [43] using a wooden toothpick positioned at the acupoint with a guide tube. The toothpick was manipulated and subjects were asked what sensations they felt and if there was any pain. During this time the piezo-stimulator tip was lowered over the acupoint. The stimulation consisted of a 2 Hz mechanical pecking to mimic EA frequency. To do this the plastic arm brace was equipped with a piezo-electric cantilever beam (Piezo Bender Q-503B, Piezo Systems, Cambridge, MA). The device was battery powered and controlled with National Instruments (NI) Labview program in combination with the 6100 DAQ card (NI) located in a laptop with Labview software. The digital signal was converted with a D/A converter and amplified (Low Cost Linear Amplifier, Piezo Systems Inc.) prior to reaching the piezo. The stimulus waveform was a single lobe from a 100 Hz half-sine wave (pulse width 5 ms). A similar "tapping" procedure has been conducted manually in acupuncture fMRI studies [13,44] and was also chosen here as it approximates control procedures used in many clinical trials

### MEG Data Collection

MEG signals were recorded with a 306-channel Vectorview MEG system (Elekta Neuromag Oy, Helsinki, Finland) housed in a custom six-layer magnetically shielded room [45]. The head position was monitored during the measurement using head position indicator coils (HPI). Locations on the subject's scalp surface and the HPI coils were digitized using a Polhemus FastTrak digitizer to allow for accurate alignment of the MEG sensor array with the subjects MRI scan. Importantly, to help minimize head motion, foam padding was used and subjects were reminded to avoid slouching and remain still. The acquisition bandwidth was 0-400 Hz with a 1200 samples/sec digitization rate. The subject's electrocardiogram (ECG) and electro-occulogram (EOG) were recorded simultaneously to control for and if necessary remove influence from physiological noise sources such as heart beat, eye blinks and eye saccading. The raw data were further processed using the signal space separation (MaxFilter, Elekta Neuromag Oy, Helsinki, Finland) to reduce the influence of magnetic fields originating from outside the subjects head.

### Structural MRI Data Collection and Cortical Surface Reconstruction

Each subject underwent an MRI scan which was co-registered with the MEG data. The anatomical MRI was used for creation of boundary element models and visualization of the cortical surface anatomy. Each subject was scanned in a Siemens Avanto 1.5 T MRI (Siemens Medical, Erlangen, Germany). Two high-resolution MPRAGE (256 × 192 matrix, 256 mm FOV,128 slices, 1.33 mm slice thickness, TE = 3.31 ms, TR = 2730 ms, TI = 1000 ms, flip = 7 deg) volumes (motion corrected and averaged offline) and a multi-echo 3D-FLASH scan (256 × 192 matrix, 256 mm FOV, 128 slices, 1.33 mm slice thickness, TE = 1.85, TR = 20 ms, 3 echoes, echo spacing = 100 μs, flip = 5deg) were acquired.

A geometrical representation of the cortical surface of each subject was obtained using procedures described previously [46,47]. First, using each subject's high-resolution 3-D, T1-weighted structural image, the cortical white matter was segmented, and the estimated border between gray and white matter was tessellated, providing a topologically correct representation of the surface with about 150,000 vertices per hemisphere. The folded surface tessellation was "inflated" in order to unfold cortical sulci, thereby providing a convenient format for visualizing MEG sources. The reconstructed surface for each subject was morphed into an average spherical representation, optimally aligning sulcal and gyral features across subjects while minimizing metric distortions and shear [47]. Compared to volumetric morphing into Talairach [48] space, this method has been found to provide better alignment across subjects of functional activation in a verbal task [49] and allows direct localization to regular gyri.

### MEG Data Processing

For evoked time-course analysis, data were low-pass-filtered at 150 Hz and separate averages of each condition were constructed for all subjects. Trials were rejected from analysis based on amplitude criteria supplemented by visual inspection for contamination by artifacts (identified as peak-to-peak amplitude (> 3,000 fT/cm in any channel) or eye blinks (> 250 mV in the EOG electrode). For each subject, S1 dipole models were fit for the 20/30 ms response for acupuncture conditions using the xfit software (Elekta Neuromag Oy, Helsinki, Finland). The location of the dipoles was projected to the cortical surface of each subject using individual MRI scans and then mapped to an average cortical surface via the spherical atlas provided by FreeSurfer [46,47]. The dipole locations for all subjects on the average cortical surface are shown in Figure 1a. Spatial filters corresponding to the P30 dipoles were utilized to generate continuous raw waveforms for each subject and condition. All subsequent analysis was performed on these waveforms. To obtain the evoked time-courses waveforms were averaged on stimulus triggers. To visualize changes in the evoked response over the course of the experiment, data were subdivided into 3 sequential windows of 5 minutes averages (Figure 1b). Significant differences between these 5 minute averages were assessed with a one-tailed paired t-test comparing the mean dipolar moment at peaks and during specific post-stimulus time intervals (Figure 1c).

Time-frequency response (TFR) analysis of the S1 source waveforms was performed to investigate (Figure 2a) induced activity during acupuncture stimulation. Induced activity describes a change in the ongoing or endogenous oscillatory activity of the brain; this activity is not phase-locked to the stimulus and cannot be seen with simple event-related analyses. In order to calculate a time-frequency distribution for these MEG data we employed a continuous wavelet transform using complex Morlet wavelets [50]. Since our study utilized very short inter-stimulus intervals (500 ms), continuous raw waveforms were wavelet transformed prior to separation into trials. This was done to prevent edge artifact contamination in the lower frequency spectrum. An average "evoked" TFR was calculated for both EA and AP conditions then subtracted from each individual trial prior to averaging for creation of the induced TFRs. The percentage change relative to the baseline mean was calculated for each frequency individually, to determine the level of ERD (event related desynchronization) and ERS (event related synchronization) [51]. To confirm that the observed induced changes were not simply the effect of a slow trending and to determine if there were time-dependent changes in the induced response, the TFR analysis was performed on sequential windows of 1 minute duration. The average percent-change values across subjects for each minute and condition are shown for a time-frequency ROI in Figure 2b.

## Authors' contributions

TW generated analysis tools and contributed significantly to data acquisition, analysis and manuscript writing. VN participated in study design, manuscript editing and was the acupuncturist for the study. NK participated in study design and manuscript editing. MV was critical in statistical analysis of data.

MH generated analysis tools and participated in manuscript editing. RD was essential to all aspects of study design, data acquisition, analysis and manuscript writing. All authors read and approved the final manuscript.

## Endnotes

^1^Translates as "obtaining qi" and traditionally refers to sensations (e.g. soreness, aching, warmth, etc.) that have been used to indicate accurate localization of an acupoint.
